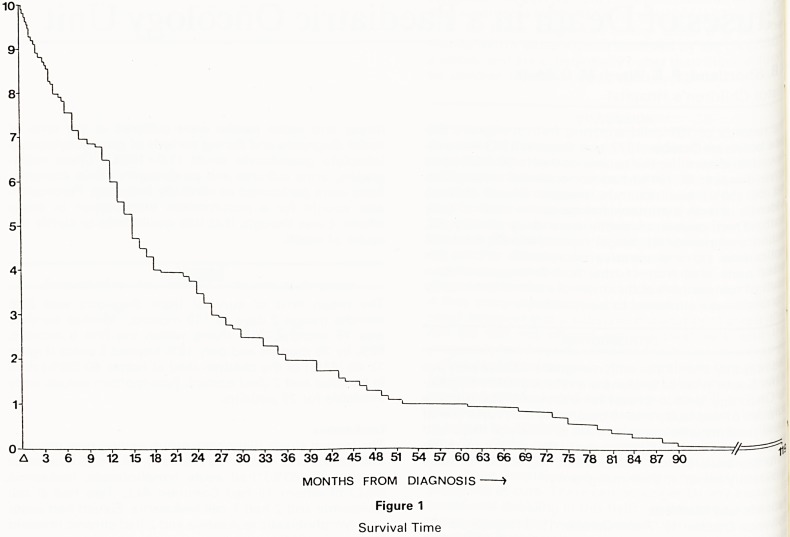# Causes of Death in a Paediatric Oncology Unit

**Published:** 1988

**Authors:** D. B. Shortland, P. S. Ward, M. G. Mott

**Affiliations:** Bristol Children's Hospital; Bristol Children's Hospital; Bristol Children's Hospital


### Causes of Death in a Paediatric Oncology Unit

D. B. Shortland; P. S. Ward; M. G. Mott
Bristol Children's Hospital

The records of 107 children dying from malignant disease between October 1977 and October 1983 were reviewed to determine the causes of death. 96 (90%) had active disease: 36 (34%) had not achieved a remission and 60 (56%) had recurrent disease. Eleven had no evidence of active primary disease at the time of their death. Their causes of death were drug toxicity (3), second malignancy (3), fungal septicaemia (2), bacterial septicaemia (2) and measles encephalitis (1). In this series none died from biochemical derangement as a result of tumour lysis at the onset of treatment, and only one death was attributed to haemorrhage.

### INTRODUCTION

The majority of children with malignant disease who live in the South West of England are referred to the Paediatric Oncology Unit in Bristol for treatment. The primary purpose of this study was to determine the proportion of these patients who die as a result of failure of treatment to control their tumour compared to the proportion dying from the toxic effects of treatment, especially in those apparently cured of their malignancy.

### Patients and Methods

Between October 1977 and October 1983 there were 337 new referrals and in the same period, 107 children died (Table 1). Their case records were examined and they were then divided into 3 groups. The first group died with evidence of active malignant disease, never having achieved a full remission. the second group had achieved a full remission but died later with recurrent disease. The third group died with no evidence of their primary malignant disease. For children in the first 2 groups we attempted to determine whether any infection present at the time of death contributed directly to mortality. In the group dying without evidence of their original malignant disease we likewise attempted to determine whether death was attributable to infection (usually as a result of the immunosuppressive effects of treatment) or to other toxic effects of treatment.

A comprehensive search was made for infection during pyrexial episodes in all children undergoing treatment. Blood samples were routinely taken for bacteriological and fungal cultures and for viral studies. Nose, throat and rectal swabs were cultured at the time of initial diagnosis and during periods of granulocytopenia (absolute granulocyte count ⟨1.0×109/L⟩. Chest radiographs, urine cultures and cerebrospinal fluid examinations were performed as clinically indicated. Permission was sought for a post-mortem examination in cases where it was thought that this would help to clarify the cause of death.

**Table 1**

**New registrations and deaths by diagnostic groups Bristol Children's Hospital October 1977–October 1983**

| | *New Registrations* | | *Deaths* | |
|---|---|---|---|---|
| | *n=* | *(%)* | *n=* | *(%)* |
| Leukaemia | 93 | (27.5) | 35 | (32) |
| Lymphoma | 53 | (16) | 12 | (11) |
| Brain Tumours | 44 | (13) | 15 | (14) |
| Bone Tumours | 26 | (7.5) | 9 | (9) |
| Nephroblastoma | 25 | (7.5) | 4 | (3) |
| Rhabdomysarcoma | 25 | (7.5) | 7 | (7.5) |
| Neuroblastoma | 17 | (5) | 12 | (11) |
| Other Tumours | 54 | (16) | 13 | (12.5) |
| | 337 | (100%) | 107 | (100%) |

### RESULTS

The mean time of survival from diagnosis was 23.6 months (range 2 days to 119 months). Median survival was 15 months, 25% dying within the first 6 months, 80% by 36 months and only 10% beyond 5 years (Figure 1). 45 (42%) of the children died at home, 60 (56%) died in hospital and 2 died abroad. Post mortem results were available for 29 patients.

### Leukaemia

The largest single diagnostic group among new referrals and deaths was leukaemia (Table 1). Of the 35 leukaemic deaths, 22 (63%) had acute lymphoblastic leukaemia (ALL) of whom 18 had Common ALL. Two had B cell leukaemia and 2 had T cell leukaemia. Eleven had acute non-lymphoblastic leukaemia and 2 had chronic myeloid leukaemia (CML).

Of the 22 deaths from ALL, 4 occurred during induction, 2 with proven gramnegative bacterial septicaemia (E. coli and Ps. aeruginosa) and 1 with generalised Aspergillosis. The other had no evidence of infection but a clinical diagnosis of brain stem haemorrhage was made. Sixteen died with recurrent disease: 4 were septicaemic at the time of death (E coli 2, Ps aeruginosa 1 and an unspecified coliform species 1); 1 developed pulmonary candidiasis terminally and a further child died from an adenovirus pneumonia; the remainder died of progressive leukaemia. Two died with no evidence of their original malignancy, the first after developing a second malignancy (AML) 8 years after treatment for ALL, and the second of measles encephalitis.

Eleven children with acute non-lymphoblastic leukemia died during the study period. Six (55%) were classified as FAB types M1 or 2 (AML). The others were promyelocytic leukaemia M3 (1), myelomonocytic leukaemia M4 (2), monoblastic leukaemia M5 (1), and erythroleukaemia M6 (1). Three died during induction and 8 of recurrent disease. Infection was present in 8 children terminally but was thought to have been directly responsible for death in only 4 (E coli 2 and Ps aeruginosa 2). A further child had radiological evidence of a lobar pneumonia but no organism was isolated from blood or sputum cultures. Three had superficial fungal infections.

Two children died with CML. A 7-month old infant with juvenile CML died without achieving remission. An 11 year old girl with adult CML died from Streptococcal meningitis while in complete remission, but on immunosuppressive therapy for chronic GVHD following a bone-marrow transplant.

### Lymphoma

Twelve children with lymphoma died, of whom 11 had non-Hodgkins lymphoma (T-cell 3, B-cell 4 and non-B

**Figure 1**

Survival Time non-T 4). Two died without achieving remission, 8 with recurrent disease and one of osteosarcoma arising as a second malignancy within the previous radiation field. Infection contributed directly to death in only 1 of those with active disease (Ps aeruginosa) and in a 2 year old child who died from systemic Aspergillosis while in complete remission after a bone-marrow transplant. Nine children died from uncontrolled spread of tumour.

### Neuroblastoma

Twelve children with neuroblastoma died. Eleven presented with stage 3 or 4 disease, of whom 5 never achieved a remission and 6 died with recurrent disease. One presented with localised ganglioneuroblastoma but relapsed with widespread disease. A full remission was achieved after re-induction with chemotherapy but some months later she developed irreversible respiratory failure as a result of bleomycin toxicity.

### Rhabdomyosarcoma

Seven children died from rhabdomyosarcoma, of whom 2 had stage 2 disease at presentation, 3 had stage 3 disease and 2 stage 4. 4 never achieved a remission and 3 had recurrent disease. All 7 died from uncontrolled disease although one had Streptococcal septicaemia terminally.

### Renal Tumours

Four children with renal tumours died. Three had stage 4 disease (pulmonary metastases) at presentation and the fourth had inoperable stage 3 disease. Two with nephroblastoma (Wilms' tumour) died from uncontrolled disease and a third from Ps. aeruginosa septicaemia while disease free but severely emaciated as a result of intensive radiation and chemotherapy. The fourth had a rhabdoid tumour (Stage 4) and died from tumour embolism at initial laparotomy, tumour thrombus extending along the renal vein and vena cava into the right atrium.

### Bone Tumours

Nine patients with bone tumours died, 4 with osteogenic sarcoma and 5 with Ewings tumour. Two had metastatic disease at presentation. Eight died of uncontrolled disease while one death was due to adriamycin cardiotoxicity and at post-mortem there was no evidence of residual tumour.

### Brain Tumours

Fifteen children with brain tumours died, 6 with medulloblastoma, 5 astrocytoma, 2 ependymoma, 1 glioblastoma multiforme and 1 unspecified glioma. Four never achieved a remission and 10 had recurrent disease. One had a fungal cerebritis at autopsy but no detectable residual tumour.

### Other Tumours

Thirteen children were included in this group. Eleven died from overwhelming disease (although one had a systemic candida infection terminally) and one after developing a second and third malignancy (sarcoma of the cheek and pinealoblastoma) following radiotherapy for bilateral retinoblastoma in infancy (ref). One child with a sacrococcygeal tumour who had metastatic disease at presentation, died from bleomycin induced respiratory failure and was disease free at the time of death.

### DISCUSSION

The pattern of new referrals was relatively constant throughout the study period. Brain tumours are under-

**Table 2**

| | *Organisms isolated at death* | *Number* |
|---|---|---|
| Gram negative bacilli | Ps aeruginosa | 6 |
| | E coli | 5 |
| | Enterobacter cloacae | 1 |
| | Unspecified coliform | 1 |
| Gram positive cocci | Lancefield group A Streptococcus | 1 |
| | Strep pneumoniae | 1 |
| Fungal Micro-organisms | Aspergillosis | 5 |
| | Candida albicans | 3 |
| Viruses | Measles | 1 |
| | Adenovirus | 1 |

represented due to referral of these children directly to Sub-Regional Neurosurgical Centres at other hospitals within the region. The pattern of deaths was broadly similar to that of new referrals but deaths due to nephroblastoma, for example, are relatively under-represented whereas those due to neuroblastoma are relatively over-represented. This reflects the recognised relatively good and poor prognosis of these two tumours respectively, as does the difference between referrals and deaths in ALL and AML.

Children with uncontrolled malignant disease commonly die in part as a result of infection but we have not considered infection to be the principal cause of death when active measures for tumour treatment had been discontinued because of resistant or progressive disease.

Infection is well recognised as a potentially lethal complication of the treatment for malignant disease (1–9). There are many factors that predispose such patients to infection. (Craft et al 1977) in a study of children receiving treatment for ALL showed that none of the 24 children who died during the study period were free from infection and infection was implicated as the main cause in all of the remission deaths. Hughes (2) showed that infection was directly responsible for 89 of 199 leukaemic deaths, the micro-organisms most commonly isolated being Ps aeruginosa, E coli, Staph. aureus and Candida albicans. Simone et al (3) reviewed children who had died during remission of leukaemia and found that 88% of the deaths were attributable to non-bacterial infection (Pneumocystis carinii, herpesviruses, cytomegalovirus and fungi). By contrast, uncontrolled disease and organ failure are the most important factors causing death during the treatment of solid tumours (11).

In the present study 96 (90%) of the children had active disease at the time of their death. 36 (34%) had not achieved a remission and 60 (56%) had recurrent disease.

Infection was present in 26 children terminally and was thought to be the principal cause of death in 19. Gram negative bacilli were isolated in 12 patients, Gram positive cocci in two, fungal micro-organisms in eight, and viruses in two (Table 2).

Infection was suspected clinically in 2 children with bronchopneumonia and lobar pneumonia though no micro-organism was isolated. Infection was thought to have contributed directly to the deaths of 15 (43%) of the 35 children with leukaemia in the study (Table 3).

Table 4 gives details of the 11 children who died without evidence of their original malignant disease. They are specially of interest since such deaths in particular are potentially preventable with current therapy. The child who died of measles' encephalitis while on maintainance treatment for ALL is a good example. It is estimated that up to 16% of patients who develop measles while on immunosuppressive therapy may develop this complication. Prophylactic immunoglobulins given to a child after contact with a suspected case of measles may lessen the chance of developing this condition (12).

Two children died from bacterial sepsis while free of malignant disease. One had had a bone-marrow transplant 12 months previously for CML. She developed an overwhelming Strep. pneumoniae septicaemia and meningitis, and died despite intensive supportive care and antibiotics. The second had a stage 3 inoperable nephroblastoma and died 2 months after diagnosis. She had had 2 months of chemotherapy before the operation. Ps aeruginosa was isolated from blood cultures during the final illness. At post-mortem there was no evidence of residual tumour. She was severely emaciated as a result of intensive chemotherapy and major surgery and this clearly contributed to the septicaemia.

Although 8 of the children in this study had evidence of fungal disease at the time of their death, only 2 were actually free of malignant disease and died as a direct result of the fungal infection. One had non-Hodgkins lymphoma but was in remission after a bone-marrow transplant and developed a severe Aspergillus pneumonia. The second had undergone surgery and radiotherapy for a medulloblastoma and was admitted to hospital 4 months after diagnosis, having developed convulsions and upper motor neurone signs. Local recurrence of the tumour was presumed and active treatment was withdrawn. At post-mortem, no evidence of residual tumour was found but cavitating lesions containing Aspergillus were identified as the lesions responsible for her neurological deterioration, a diagnosis totally unsuspected

**Table 3**

| | | *Timing of Death* | | | | *Cause of Death* | | | |
|---|---|---|---|---|---|---|---|---|---|
| | *n* | *Induction* | *Recurrent disease* | *Remission* | *Active disease* | *Infection* | *Intracranial haemorrhage* | *Drug toxicity* | *Second malignancy* |
| Leukaemia | 35 | 8 | 24 | 3 | 18 | 15 | 1 | | 1 |
| Lymphoma | 12 | 2 | 8 | 2 | 9 | 2 | | | 1 |
| Brain Tumours | 15 | 4 | 10 | 1 | 14 | 1 | | 1 | |
| Bone Tumours | 9 | 0 | 8 | 1 | 8 | | | | |
| Neuroblastoma | 12 | 5 | 6 | 1 | 11 | | | 1 | |
| Nephroblastoma | 4 | 3 | 0 | 1 | 3 | 1 | | | |
| Rhabdomyosarcoma | 7 | 4 | 3 | 0 | 7 | | | | |
| Others | 13 | 10 | 1 | 2 | 11 | | | 1 | 1 |

**Table 4**

**Deaths of children in remission**

| *Patients* | *Diagnosis* | *Cause of Death* |
|---|---|---|
| 1 | All | Measles encephalitis |
| 2 | CML | Pneumococcal meningitis (post BMT) |
| 3 | Nephroblastoma | Pseudomonas septicaemia |
| 4 | NHL(T) | Aspergillosis (post BMT) |
| 5 | Medulloblastoma | Aspergillus cerebritis |
| 6 | Osteosarcoma | Adriamycin cardiotoxicity |
| 7 | Neuroblastoma | Bleomycin pneumonitis |
| 8 | Malignant teratoma | Bleomycin pneumonitis |
| 9 | All | Second malignancy |
| 10 | Retinoblastoma | Second malignancy |
| 11 | NHL (B) | Second malignancy |

pre-mortem. Fungal infections are a well recognised complication of immunosuppressive treatment and Aspergillus is the most frequent invasive organism. In the study by Mirsky and Cuttner (7) 28% of the patients who died from acute leukaemia had evidence of severe fungal infection. These authors and others have emphasised the difficulty of making a clinical or laboratory diagnosis of systemic fungal infection before death.

The systemic side-effects of chemotherapy are well recognised complications of the treatment of malignant disease. In the present study 3 children died as a direct result of such toxicity and a fourth who died with relapsed non-Hodgkins lymphoma had evidence of severe methotrexate encephalopathy at post-mortem. Bleomycin lung toxicity was responsible for the deaths of 2 children aged 2 and 4 years. The frequency of this complication is thought to be related to the cumulative dose of bleomycin given and it is estimated that around 1% of patients will develop a fatal pulmonary fibrosis (13). A 14 year old with osteosarcoma of the femur died 14 months after diagnosis from adriamycin induced cardiomyopathy at a cumulative dose of 500 mg per sq meter. No evidence of residual tumour was found at post-mortem. Cardiotoxicity is the most feared complication of adriamycin treatment and is related to the total dose given (14). The incidence of this complication may be as high as 30% when the total dose exceeds 550 mg per sq meter (15).

The development of a second malignancy after treatment for childhood cancer is well documented (16). One child who had bilateral familial retinoblastoma in infancy treated with radiotherapy (4,000 rads) went on to develop pinealoblastoma, so-called trilateral retinoblastoma and sarcoma of the cheek 4 years later. A 13 year old developed AML after achieving an 8 year remission of ALL, and a child cured of abdominal NHL by radiation and chemotherapy developed osteosarcoma in the radiation field.

Cerebral haemorrhage is a potentially fatal complication of treatment during periods of profound thrombocytopenia. This diagnosis was suspected clinically in 1 infant dying during attempted induction of remission of ALL while active disease was still present in his bonemarrow. When it became apparent that treatment was not controlling his disease, the medical staff discussed with his parents the option of nursing him at home. His was the only death attributed to haemorrhage in this series and none were attributed to biochemical derangements due to tumour lysis at the onset of treatment.

### CONCLUSIONS

Most of the children who died had active malignant disease at the time of death. The failure of present treatment regimens to eradicate the tumour remains the single most important cause of death in children with cancer. Infection is frequently present terminally in such children but is more important as the primary cause of death in children on immunosuppressive treatment at a stage when they may be free from malignant disease. Infection was frequently responsible for the deaths which occurred during induction of remission in the present study. Although these children had evidence of active disease, it is impossible to predict how many would have achieved a long term remission, had the infection been successfully treated. Fungal infections are less common than bacterial, but pose a particularly difficult problem for diagnosis and treatment. Viral infections were responsible for the deaths of only 2 children.

The toxic side-effects of chemotherapeutic agents are well documented and all children on treatment have to be monitored closely so that their treatment can be adjusted before irreversible organ damage is sustained. Children who have been successfully treated for malignant disease are known to be at increased risk of developing a second malignancy and must be closely monitored to detect this complication while it is still potentially treatable.

### REFERENCES

1. CRAFT, A. W., REID, M. M., BRUCE, E., KERAHAN, J. and ARDNER, P. S. G. (1977) Role of infection in the death of children with acute lymphoblastic leukaemia. *Arch.Dis. Child* **52**, 752–757.
2. HUGHES, W. T. (1971) Fatal infections in childhood leukaemia. *Am.J.Dis.Child* **122**, 283–287.
3. SIMONE, J. V., HOLLAND, E. and JOHNSON, W. (1972) Fatalities during remission of childhood leukaemia. *Blood* **39**, 759–769.
4. HUGHES, W. T., SMITH, D. R. (1973) Infection during induction of remission in acute lymphocytic leukaemia. *Cancer* **31**, 1008–1014.
5. KOSMIDOS, H. V., LUSHER, J. M. SHOPE, T. C., RAVINDRANATH, Y., DAJINI, A. S. (1980) Infections in leukaemic children: A prospective analysis. *J.Peds.* **96**, 814–819.
6. FEIGIN, R. D., SHEARER, W. T. (1975) Opportunistic infections in children – ii: In the compromised host. *J. Peds.* **87**, 677–694.

7. MIRSKY, H. S., CUTTNER, J. (1972) Fungal infection in acute leukaemia. *Cancer* **30**, 348–352.
8. PIZZO, P. A. (1981) Infectious complications in the child with cancer ii management of specific infectious organisms. *J.Peds.* **98**, 513–523.
9. VIOLA, M. V. (1967) Acute leukaemia and infection. *JAMA* **201**, 923–926.
10. STRAUSS, R. R., PAUL, B. B., JACOBS, A. A., SIMMONS, C., SHARRA, A. J. (1970) The metabolic and phagocytic activities of leukocytes from children with acute leukaemia. *CancerRes.* **30**, 480–488.
11. PEREZ-MESA, C. M. (1983) Causes of death of patients with solid tumours. *Cancer Treatment Symposia* **2**, 171–176.
12. Measles encephalitis during immunosuppressive treatment. (1976) *BMJ* **1**, 1552.
13. ALTMAN, A. J. and SCHWARTZ, A. D. Cancer chemotherapy "Malignant Diseases of Infancy, Childhood and Adolescence". 1983 W. B. Saunders Company 59–95.
14. RAMOS, A., MEYER, R., KORFHAGEN, J., YUEN WONG, K., KAPLAN, S. (1976) Echocardiographic evaluation of Adriamycin cardiotoxicity in children. *Cancer Treatment Reports* **60**, 1281–1286.
15. LEFRAK, E. A., PITHA, J., ROSENHEIM, S. (1973) A clinicopathologic analysis of adriamycin cardiotoxicity. *Cancer* **32**, 302–314.
16. KINGSTON, J. E. (1985) Second primary tumours. *Arch.Dis. Child.* **60**, 695–697.

## Figures and Tables

**Figure 1 f1:**